# Prehospital Diagnosis and Treatment of Patients With Acute Heart Failure

**DOI:** 10.7759/cureus.25866

**Published:** 2022-06-12

**Authors:** Michael Supples, Katelyn Jelden, Jenna Pallansch, Frances M Russell

**Affiliations:** 1 Emergency Medicine, Indiana University School of Medicine, Indianapolis, USA

**Keywords:** treatment, shortness of breath, acute heart failure, diagnosis, prehospital

## Abstract

Introduction

Early diagnosis and optimization of heart failure therapies in patients with acute heart failure (AHF), including in the prehospital setting, is crucial to improving outcomes. However, making the diagnosis of AHF in the prehospital setting is difficult. The goal of this study was to evaluate the accuracy of prehospital diagnosis (AHF versus not heart failure [HF]) in patients with acute dyspnea when compared to final hospital diagnosis.

Methods

We conducted a retrospective study of adult patients transported by emergency medical services (EMS) with a primary or secondary complaint of shortness of breath. Patients were identified through an EMS electronic database (ESO) and matched to their hospital encounter. ESO was reviewed for prehospital diagnosis and management. Hospital electronic medical records were reviewed to determine final hospital diagnosis, management in the emergency department and hospital, disposition, and length of stay. The primary outcome compared prehospital diagnosis to final hospital diagnosis, which served as our criterion standard.

Results

Of 199 included patients, 50 (25%) had a final diagnosis of AHF. Prehospital paramedic sensitivity and accuracy for AHF were 14% (7/50; confidence interval [CI] 0.06-0.26) and 77% (CI 0.70-0.82), respectively. In the 50 patients with AHF, 14 (28%) received nitroglycerin in the prehospital setting, while 27 (54.0%) patients were inappropriately treated with albuterol.

Conclusion

Prehospital paramedics had poor sensitivity and moderate accuracy for the diagnosis of AHF. A small percentage of patients ultimately diagnosed with AHF had HF therapy initiated in the prehospital setting. This data highlights the fact that AHF is difficult to diagnose in the prehospital setting and is commonly missed.

## Introduction

Acute heart failure (AHF) remains a pervasive cause of morbidity and mortality with a rising prevalence in the United States [1]. Annually, AHF is the primary diagnosis in over one million United States emergency department visits and principal cause for more than 80,000 deaths [2]. In 2012, over 30 billion dollars were spent on HF alone, with over 50% of the costs being consumed by AHF hospitalizations [3]. Despite enormous health care costs and an increasing prevalence, there remains little evidence to inform acute management of these patients, leading to suboptimal care with poor outcomes [3]. Outcomes for patients with AHF have remained relatively unchanged over the last decade [4-6], with an absolute mortality rate of about 50% within five years of diagnosis [7]. Poor outcomes can be attributed to lack of Class 1, Level of Evidence: A recommendations, high practice variation, misdiagnosis, delayed diagnosis, and delayed treatment [3].

Emergency medical services (EMS) transports for acute dyspnea are common. When looking at non-trauma-related reasons for transport to a hospital, one in 10 patients has dyspnea [8]. In the prehospital setting, diagnosis of AHF is challenging as many signs and symptoms of AHF overlapping with other etiologies of dyspnea [9]. Available history is often limited, and diagnostic tools such as labs and radiography are not readily available when compared to the hospital setting.

Prior studies examining prehospital diagnosis of AHF in patients with a chief complaint of dyspnea have found it to be challenging to make a diagnosis of AHF [10,11]. Other data have shown administration of HF therapies in the prehospital setting to patients with AHF is low [11,12].

AHF necessitates a prompt and accurate diagnosis leading to judicious medication administration in the prehospital setting. The goal of this study was to determine the accuracy of prehospital paramedic diagnosis for patients being transported to the hospital by EMS for acute dyspnea when compared to final hospital diagnosis. Secondarily, we evaluated accuracy of prehospital treatment based on patient final diagnosis.

## Materials and methods

Study design and participants

This was a retrospective study of adult patients transported by a single urban EMS agency to a single hospital system consisting of 15 hospitals with emergency departments, of which three receive regular adult EMS transports from the target agency. The combined emergency department annual volume is over 160,000 patient visits. This study was conducted from October 2018 to December 2018, and it was approved by the Institutional Review Board (#12749) with a waiver of informed consent.

Study protocol

We included all adult patients (>18 years old) with suspected dyspnea transported by EMS. We excluded patients with a final hospital diagnosis, based on chart review, that was not a cardiac or pulmonary etiology of dyspnea (i.e., alcohol withdrawal, anemia, anxiety, etc.).

Using the EMS electronic health record, ESO (Austin, TX), we identified sequential adult patients with EMS encounters for dyspnea. In an attempt to include all patients with dyspnea transported by EMS during this time frame, we included patients with a cardiac or pulmonary primary or secondary EMS impression documented in ESO, see Table 1. The following variables were extracted automatically from ESO to a REDCap database: incident date and time, transport destination, demographics including age, sex, race and ethnicity, and initial vital signs (blood pressure, heart rate, respiratory rate, pulse oximetry, and Glasgow Coma Scale), EMS on-scene time and EMS transport time, primary and secondary impression, airway interventions (oxygen, non-invasive positive pressure ventilation [NIPPV], supraglottic airway insertion, endotracheal tube insertion), and medication administration (albuterol, nitroglycerin). Furosemide data was not collected, as this medication was not an available prehospital medication.

**Table 1:** Emergency Medical Services Recorded Primary and Secondary Impression for Inclusion

Emergency Medical Services Recorded Primary and Secondary Impression for Inclusion
Acute heart failure (AHF)
Acute respiratory distress (dyspnea)
Asthma
Chronic obstructive pulmonary disease (COPD)
Edema
Generalized edema
Hypertensive crisis
Hyperventilation
Pitting edema
Pneumonia
Pulmonary edema
Respiratory arrest
Respiratory failure
Shortness of breath

Patients were matched to their in-hospital encounter by name and date of service. The following variables were automatically extracted from the hospital electronic medical records: disposition (admit or discharge), admission to a progressive care unit or intensive care unit (ICU), hospital length of stay, ICU length of stay, in-hospital mortality, and medications administered during hospital encounter (albuterol, furosemide, nitroglycerin, intravenous fluids), supplemental oxygen administration, and NIPPV.

Medical record review

Hospital electronic medical records were reviewed by one of four emergency medicine physicians to determine patient inclusion/exclusion in the study, final hospital diagnosis, and to determine if the patient was intubated or had an advanced airway placed in the hospital. Physicians included two faculty with extensive research experience, one fellow and one senior resident. To ensure standardization of chart review, prior to beginning chart review, all reviewers read and utilized guidelines from Gilbert et al. [13]. Additionally, all four reviewers went over the data collection form together. Then 10 records were reviewed by all four reviewers with the following kappa statistics: study inclusion (k=0.89), diagnosis (k=0.78), intubation (k=1.0). Any discrepancies were reviewed by the group.

Outcomes

The primary outcome compared prehospital paramedic diagnosis to final hospital diagnosis, which served as our criterion standard. If a paramedic documented "acute heart failure", "heart failure", or "pulmonary edema" as the primary or secondary impression in ESO then this counted as a prehospital diagnosis of acute heart failure. If a vague impression was listed, such as "acute respiratory distress" or "dyspnea", this was counted as a non-heart failure diagnosis. If a patient was found to have a multifactorial cause for their dyspnea (e.g., AHF and COPD together) then they were included in the group of patients with AHF as the final diagnosis. Secondarily, we evaluated the rate of initiation of AHF therapies in patients with a final hospital diagnosis of AHF.

Statistical analysis

We used SAS 9.4 (Cary, NC) for all analysis. Interrater reliability for the chart review was estimated with kappa statistics calculated for 10 case reviews performed independently by all reviewers. We performed descriptive statistics with a mean (standard deviation) for parametric continuous variables and median (interquartile range) for nonparametric continuous variables. Using final hospital diagnosis as the criterion standard for AHF, we calculated test characteristics (sensitivity, specificity, and accuracy) of EMS's primary and secondary impression of AHF or pulmonary edema via a 2x2 table. We used logistic regression with stepwise forward conditional inclusion with p<0.10 to identify factors associated with a missed diagnosis of AHF. We considered p<0.05 to be statistically significant.

## Results

Out of 266 cases reviewed, 199 (75%) patients met inclusion/exclusion criteria and were included in the analysis. One hundred and seventeen (58%) patients were female, the median age was 70 (IQR 49-72) years, and 114 (57%) identified as African American, see Table 2 for patient demographics. Forty (20%) and 137 (69%) patients had a past medical history of heart failure and chronic obstructive pulmonary disease (COPD), respectively. Most patients were admitted (149/199, 75%), with over half of the admitted patients being placed in an intermediate or intensive care unit (79/149, 53.0%). A higher percentage of patients with AHF received NIPPV prehospital (12/50, 24.0% versus 12/149, 8.1%; p=0.010) and in-hospital (24/50, 48% versus 39/110, 26.2%; p=0.004). AHF was the final hospital diagnosis in 50 (25%) of the 199 patients.

**Table 2:** Patient demographics with and without AHF AHF = acute heart failure; AMS = altered mental status, COPD = chronic obstructive pulmonary disease, CPAP = continuous positive airway pressure, HR = heart rate, IQR = interquartile range, SBP = systolic blood pressure

Patient demographics with and without AHF			
	Final Hospital Diagnosis		
	AHF	Not AHF	p-value
	n=50	n=149	
Age, Median (IQR)	63 (57-71)	58 (50-69)	0.011
Female, n(%)	24 (48.0%)	91 (61.1%)	0.105
Race/Ethnicity, n(%)			0.110
Asian	1 (2.0%)	0	
African American	28 (56.0%)	85 (57.1%)	
Hispanic	1 (2.0%)	0	
White	20 (40.0%)	64 (42.9%)	
Hospital NIPPV, n(%)	24 (48.0%)	39 (26.2%)	0.004
Hospital Advanced Airway, n(%)	3 (6.3%)	12 (9.0%)	0.550
Admitted to Hospital, n(%)	42 (84.0%)	103 (69.1%)	0.041
Admitted to IICU or ICU, n(%)	30 (60.0%)	47 (31.5%)	<0.001
Died in Hospital, n(%)	0	6 (4.0%)	0.150
Initial Prehospital Vital Signs, n(%)			
Hypoxic (SpO2 <92%)	26 (52.0%)	67 (45.0%)	0.388
Hypotensive (SBP <90 mmHg)	14 (28.0%)	50 (33.6%)	0.467
Hypertension (SBP >160 mmHg)	9 (18.0%)	19 (12.8%)	0.356
Bradycardic (HR <60 bpm)	17 (34.0%)	51 (34.2%)	0.977
Tachycardic (HR >100bpm)	16 (32.0%)	42 (28.2%)	0.608
AMS (GCS<15)	18 (36.0%)	52 (34.9%)	0.888
Prehospital Treatments, n(%)			
Bronchodilator	27 (54.0%)	91 (61.1%)	0.378
Corticosteroid	5 (10.0%)	22 (14.78%)	0.395
Nitroglycerin	14 (28.0%)	4 (2.7%)	<0.001
Oxygen	33 (66.0%)	85 (57.1%)	0.265
CPAP	12 (24.0%)	12 (8.1%)	0.010
Prehospital Impression, n(%)			
COPD	4 (8.0%)	19 (12.8%)	0.363
Acute Heart Failure	7 (14.0%)	3 (2.0%)	0.001

Of the 50 patients with a final diagnosis of AHF, seven were correctly identified by EMS, indicating a sensitivity of 14% (CI 0.06-0.26). The specificity and diagnostic accuracy of EMS for AHF were 98% (CI 0.94-0.99) and 77% (CI 0.70-0.82), respectively. See Table 3. 

**Table 3:** Final hospital diagnosis versus EMS impression of AHF AHF = acute heart failure

		Hospital AHF Diagnosis		
		Yes	No	
EMS AHF Impression	Yes	7	3	189
	No	43	146	10
		50	149	199

Of the 50 patients with AHF, 14 (28%) received nitroglycerin, and 27 (54.0%) received albuterol in the prehospital setting. 

A logistic regression model was constructed to estimate the odds of failing to diagnose AHF based on age, sex, race, and abnormal vital signs (hypoxia, hypotension, hypertension, bradycardia, tachycardia, altered mental status). In a single variable analysis, no factors were significantly associated with a missed diagnosis. We evaluated multiple variable logistic regression using a forward stepwise procedure (p<0.10) and found no variables to be statistically significant. See Table 4.

**Table 4:** Univariate odds of not identifying AHF by demographics and initial vital sign abnormalities. AHF = acute heart failure; AMS = altered mental status, CI = confidence interval, HR = heart rate, SBP = systolic blood pressure

Univariate odds of not identifying AHF by demographics and initial vital sign abnormalities.			
	Odds Ratio	95% CI	p-value
Age	1.003	(0.99 - 1.02)	0.670
Sex			
Male	referent		
Female	1.10	(0.56 - 2.17)	0.785
Race/Ethnicity, n (%)			0.110
White	referent		
African American	0.91	(0.45 - 1.83)	0.929
Initial Prehospital Vital Signs			
Hypoxia (SpO2 <92%)	1.31	(0.67 - 2.58)	0.428
Hypotension (SBP <90 mmHg)	0.93	(0.45 - 1.92)	0.837
Hypertension (SBP >160 mmHg)	1.02	(0.39 - 2.69)	0.973
Bradycardia (HR <60 bpm)	1.08	(0.53 - 2.20)	0.828
Tachycardia (HR >100bpm)	1.20	(0.58 - 2.47)	0.627
AMS (GCS<15)	1.16	(0.58 - 2.34)	0.672

## Discussion

Being able to accurately discern AHF from other etiologies of dyspnea and initiate disease-specific treatment in the prehospital setting is crucial as this has a direct impact on patient prognosis [14-17]. Increasingly “time to therapy” is thought to be of great benefit in patients with AHF, similar to patients with acute coronary syndrome [18-20]. In this study, we found paramedics only correctly diagnosed seven out of 50 patients (14%) with AHF, and of these patients, only one-quarter were started on AHF therapies before hospital arrival. 

These results are similar to prior studies evaluating the prehospital diagnostic accuracy of AHF and initiation of treatment. A single-center retrospective study on 122 patients by Pozner et al. found that prehospital diagnosis compared to emergency department diagnosis was 77% correct [10]. Pan et al. studied 330 patients and also found that AHF was difficult to diagnose in the prehospital setting. Additionally, they found that 49% of patients with AHF were not treated with furosemide, and greater than one-third of patients given furosemide in the prehospital setting were found not to have AHF [11]. In a retrospective study from the Helsinki metropolitan area on 100 patients transported by EMS, the use of prehospital medications was low, with only one-third of patients receiving therapy before hospital arrival [12].

Our study is innovative as we also assessed for factors associated with a missed diagnosis of AHF. Unfortunately, we did not identify any factors using univariate and multivariate analysis that was associated with the missed diagnosis. Although this was not the primary aim of our paper, this may be an area of further research to address misdiagnosis.

Forty-three patients (86%) with AHF were misdiagnosed with an alternative or vague etiology, including acute respiratory distress (25; 58%), shortness of breath (11; 26%), and COPD (4; 9%). This is important as diagnostic momentum can lead to cognitive errors and anchoring bias where a diagnostic label can be attached to a patient [21]. This can cause a subsequent provider to accept a prior diagnosis that may be incorrect. This, in turn, leads to further delays in diagnosis and treatment. Alternatively, vague etiologies may be indicative of an inability to discriminate between different diseases (e.g., AHF vs. COPD). 

Of the 50 patients with a final diagnosis of AHF, only 14 (28%) were treated with nitroglycerin, and 12 (24%) were treated with NIPPV in the prehospital setting. In the hospital, an additional 12 (24%) patients with AHF required NIPPV. A large portion of patients (36/50, 72%) did not receive AHF therapies that were available to administer in the prehospital setting. This is of concern as we know earlier treatment is associated with improved outcomes [14-16], and delayed therapy is linked to higher mortality [17,22]. Four patients that did not have a prehospital diagnosis of AHF received nitroglycerin to treat chest pain.

In addition, over half of the patients with a final diagnosis of AHF were treated inappropriately with a bronchodilator (albuterol). Although we did not assess patient outcomes as we were not powered to do so, prior data has shown that prehospital and emergency department bronchodilator administration in patients with AHF is associated with an increased risk of in-hospital mechanical ventilation, intensive care unit admission, and longer hospital length of stays [23]. 

While specificity was high at 98%, this is unlikely a reflection of diagnostic accuracy and more likely explained by a population with a high prevalence of an alternative etiology for shortness of breath and reflects overdiagnosis of COPD and asthma in patients with shortness of breath.

Limitations

This study has several limitations that may affect its generalizability. It is a retrospective study that assessed a single EMS agency and a single hospital system. However, the hospital system included a broad spectrum of levels of care, from critical access hospitals to an urban academic trauma center, which should limit this as a source of selection bias. The small sample size of 50 AHF patients increases the chance of error. 

A small portion of our patients had multifactorial sources of dyspnea, specifically AHF combined with a non-cardiogenic source, such as COPD. Similarly, several of our patients with isolated diagnoses of AHF had underlying pulmonary conditions like COPD or asthma. Administration of albuterol in these patients may not be harmful, as suggested by prior literature [23]; however, further research is needed to clarify the effects of albuterol given to patients with AHF but also with underlying COPD. 

Finally, the acuity of our patients compared to more general samples is unclear. No patients died during hospitalization in our study, whereas the in-hospital mortality typically seen in patients with acute heart failure exacerbations is 3% [24]. Additionally, the typical intubation rate is 3%; however, 6% of our patients received an advanced airway [25]. 

## Conclusions

This retrospective study suggests that paramedics had poor sensitivity and moderate accuracy for the diagnosis of AHF. Prehospital administration of HF therapies was low. This data further demonstrates the fact that AHF is difficult to diagnose in the prehospital setting and is commonly missed. Future research should focus on ways to improve diagnostic and therapeutic strategies for patients with AHF in the prehospital setting.
